# Supplementary material for: Association between obesity and mortality from hematological malignancies among Japanese adults: The Japan Collaborative Cohort study
Source: PLoS One. 2025 Oct 30;20(10):e0335750. doi: 10.1371/journal.pone.0335750 (PMC12574875; doi:10.1371/journal.pone.0335750)
Supplement: S1 Table — (PDF) [file pone.0335750.s001.pdf]

		All hematological malignancies (C81-C96) HR (95% CI)	Lymphoma (C81-C86) HR (95% CI)	Multiple myeloma (C90) HR (95% CI)	Leukemia (C91-C95) HR (95% CI)	Myeloid Leukemia (C92) HR (95% CI)
Model 2						
BMI	Underweight	1.15 (0.79, 1.68)	1.06 (0.59, 1.92)	0.79 (0.32, 1.96)	1.60 (0.90, 2.85)	1.08 (0.47, 2.50)
	Normal-weight	ref.	ref.	ref.	ref.	ref.
	Overweight	0.93 (0.74, 1.18)	0.87 (0.60, 1.27)	0.81 (0.48, 1.37)	1.06 (0.72, 1.57)	0.80 (0.47, 1.38)
	Obese	1.77 (1.02, 3.09)	0.65 (0.16, 2.63)	2.84 (1.15, 7.02)	2.47 (1.08, 5.62)	3.87 (1.68, 8.90)
Sex	Female	ref.	ref.	ref.	ref.	ref.
	Male	1.74 (1.45, 2.08)	2.08 (1.57, 2.76)	1.30 (0.88, 1.90)	1.65 (1.21, 2.24)	2.18 (1.48, 3.21)
Age	40s	ref.	ref.	ref.	ref.	ref.
	50s	2.19 (1.63, 2.94)	3.43 (2.04, 5.77)	1.67 (0.89, 3.12)	1.70 (1.09, 2.67)	1.77 (0.98, 3.17)
	60s	3.46 (2.60, 4.61)	5.30 (3.18, 8.85)	3.43 (1.91, 6.17)	2.36 (1.51, 3.70)	2.89 (1.64, 5.10)
	70s	5.41 (3.86, 7.57)	8.14 (4.56, 14.54)	5.96 (3.03, 11.74)	3.42 (1.96, 5.96)	4.04 (2.00, 8.17)
Model 3						
BMI	Underweight	1.14 (0.78, 1.66)	1.04 (0.57, 1.88)	0.76 (0.30, 1.92)	1.61 (0.90, 2.89)	1.11 (0.48, 2.61)
	Normal-weight	ref.	ref.	ref.	ref.	ref.
	Overweight	0.94 (0.74, 1.20)	0.90 (0.62, 1.32)	0.80 (0.47, 1.36)	1.06 (0.72, 1.58)	0.80 (0.46, 1.38)
	Obese	1.78 (1.02, 3.11)	0.67 (0.16, 2.73)	2.75 (1.09, 6.94)	2.47 (1.07, 5.69)	3.89 (1.66, 9.11)
Sex	Female	ref.	ref.	ref.	ref.	ref.
	Male	1.40 (1.02, 1.90)	1.92 (1.18, 3.11)	1.24 (0.62, 2.46)	1.02 (0.60, 1.73)	1.30 (0.66, 2.55)
Age	40s	ref.	ref.	ref.	ref.	ref.
	50s	2.20 (1.63, 2.97)	3.61 (2.13, 6.13)	1.48 (0.78, 2.83)	1.72 (1.08, 2.74)	1.80 (0.98, 3.29)
	60s	3.48 (2.54, 4.76)	6.14 (3.54, 10.64)	2.59 (1.35, 4.95)	2.36 (1.43, 3.90)	3.04 (1.62, 5.70)
	70s	5.41 (3.66, 7.98)	10.07 (5.21, 19.45)	4.19 (1.90, 9.23)	3.34 (1.73, 6.45)	4.39 (1.94, 9.93)
Living area of childhood	Large cities	ref.	ref.	ref.	ref.	ref.
	Other cities	0.91 (0.54, 1.54)	0.90 (0.41, 2.00)	0.53 (0.11, 2.46)	0.94 (0.40, 2.20)	1.03 (0.38, 2.83)
	Rural areas/Islands or remote areas	0.90 (0.66, 1.21)	0.70 (0.46, 1.07)	1.43 (0.73, 2.79)	0.91 (0.54, 1.52)	0.96 (0.51, 1.80)
	Others	1.51 (0.96, 2.37)	1.36 (0.69, 2.67)	1.70 (0.58, 4.96)	1.63 (0.78, 3.41)	2.25 (0.95, 5.35)
Educational attainment	<10 years	ref.	ref.	ref.	ref.	ref.
	<13 years	0.86 (0.69, 1.08)	0.91 (0.65, 1.26)	1.13 (0.70, 1.85)	0.69 (0.46, 1.03)	0.81 (0.50, 1.32)
	<16 years	1.03 (0.74, 1.45)	0.87 (0.50, 1.52)	1.40 (0.69, 2.85)	1.00 (0.58, 1.75)	0.96 (0.47, 1.93)
	≥16years	0.80 (0.42, 1.54)	0.70 (0.25, 1.94)	0.94 (0.21, 4.19)	0.87 (0.32, 2.38)	0.83 (0.24, 2.85)
Current job at the baseline	Employed/Part-time	ref.	ref.	ref.	ref.	ref.
	Self-employed	1.12 (0.86, 1.45)	0.92 (0.62, 1.37)	1.63 (0.86, 3.11)	1.19 (0.78, 1.84)	1.15 (0.69, 1.93)
	Homemaker	0.88 (0.61, 1.27)	0.77 (0.43, 1.38)	1.93 (0.88, 4.22)	0.69 (0.36, 1.33)	0.70 (0.31, 1.56)
	Unemployed	0.99 (0.70, 1.38)	0.72 (0.43, 1.21)	2.35 (1.10, 5.01)	0.86 (0.48, 1.54)	0.69 (0.33, 1.42)
	Others	0.70 (0.42, 1.17)	0.25 (0.08, 0.82)	2.53 (1.05, 6.10)	0.69 (0.29, 1.65)	0.45 (0.13, 1.54)
Drinking status	Current drinker	ref.	ref.	ref.	ref.	ref.
	Former drinker	1.00 (0.60, 1.68)	1.21 (0.58, 2.51)	0.85 (0.27, 2.73)	0.74 (0.27, 2.01)	0.81 (0.25, 2.64)
	Non-drinker	0.98 (0.78, 1.24)	1.20 (0.84, 1.71)	0.86 (0.51, 1.42)	0.85 (0.57, 1.26)	0.89 (0.54, 1.49)
Smoking status	Current smoker	ref.	ref.	ref.	ref.	ref.
	Former smoker	0.72 (0.53, 0.98)	0.50 (0.31, 0.81)	0.91 (0.47, 1.78)	0.97 (0.59, 1.59)	1.20 (0.69, 2.11)
	Non-smoker	0.72 (0.54, 0.97)	0.67 (0.43, 1.04)	0.86 (0.44, 1.68)	0.72 (0.43, 1.18)	0.71 (0.38, 1.33)
Exercise habits of the week	Minimal	ref.	ref.	ref.	ref.	ref.
	1–2 hours	1.14 (0.86, 1.50)	1.24 (0.82, 1.87)	0.81 (0.41, 1.60)	1.26 (0.79, 2.00)	1.19 (0.68, 2.09)
	3–4 hours	0.88 (0.58, 1.32)	0.76 (0.37, 1.52)	0.63 (0.24, 1.66)	1.13 (0.56, 2.28)	1.05 (0.46, 2.42)
	≥5 hours	1.23 (0.84, 1.79)	1.25 (0.69, 2.24)	0.67 (0.25, 1.79)	1.68 (0.93, 3.02)	1.62 (0.81, 3.24)
Walking habits of the day	Minimal	ref.	ref.	ref.	ref.	ref.
	Around 30 min	1.07 (0.72, 1.60)	0.99 (0.48, 2.05)	1.18 (0.51, 2.74)	1.00 (0.50, 2.00)	0.97 (0.41, 2.30)
	30 min to 1 hour	1.19 (0.79, 1.80)	1.61 (0.81, 3.20)	0.84 (0.36, 1.94)	0.98 (0.50, 1.90)	1.14 (0.51, 2.53)
	≥1 hour	1.08 (0.75, 1.55)	1.32 (0.72, 2.45)	0.81 (0.39, 1.70)	1.05 (0.57, 1.92)	1.05 (0.50, 2.18)
