# Supplementary material for: Association between obesity and mortality from hematological malignancies among Japanese adults: The Japan Collaborative Cohort study
Source: PLoS One. 2025 Oct 30;20(10):e0335750. doi: 10.1371/journal.pone.0335750 (PMC12574875; doi:10.1371/journal.pone.0335750)
Supplement: Attachment — Submitted filename: response to reviewers_20250826.docx [file pone.0335750.s003.docx]

Revision Note

We are most grateful to you and the reviewers for the comments on our manuscript. These have greatly helped us to improve our manuscript.　We have revised our manuscript based on the constructive suggestions of the reviewers.

Please note, in the responses below, we have included extra citations that are used only for explaining our rationale to the reviewers and are not included in the main manuscript. Such response-only references are indicated in the format “(R1, R2……)”.

The following are our responses to the reviewer 1 and 2. Our responses are in blue, and the changes are in italics. Also, the changes are shown in red in the revised manuscript.

Reviewer 1

1. As the authors used mortality rather than incidence as the primary outcome, it is important to acknowledge that the observed associations may reflect not only disease risk but also differences in survival. For hematologic malignancies with relatively favorable prognosis, such as certain subtypes of lymphoma, associations with BMI may be attenuated or obscured when mortality is used as the endpoint. Although the discussion briefly touches upon this point, the authors should more clearly elaborate on the implications of using mortality as the outcome.

A more explicit consideration of this limitation would help contextualize the null findings for lymphoma and strengthen the overall interpretation. Furthermore, given that the JACC Study likely possesses incidence data based on previous publications, a discussion of whether the association between BMI and hematologic malignancies would differ if incidence (rather than mortality) were used as the outcome would greatly enhance the depth and value of the manuscript.

Author response

We thank the reviewer for this important and insightful comment. We appreciate the opportunity to clarify the rationale behind our choice of mortality as the primary outcome in this study.

The primary objective of our research was to investigate the association between BMI and mortality from hematological malignancies. Mortality is a critical endpoint because it reflects not only the risk of developing the disease but also the integrated biological and clinical consequences after diagnosis, including treatment response, toxicity, recurrence, and survival. From a public health perspective, reducing cancer mortality is a major goal, as emphasized in Japan’s national cancer control policy [R1]. Thus, studying mortality provides essential evidence to guide strategies aimed at improving long-term outcomes for patients and society.

We agree that, for hematologic malignancies with relatively favorable prognoses such as lymphoma, the use of mortality as an outcome may attenuate or obscure associations with BMI. In our study, the lack of a statistically significant association between BMI and lymphoma mortality may indeed be partly attributable to the higher survival rate of lymphoma compared with other hematologic malignancies. To address this point, we have now added a sentence in the Discussion noting that differences in survival rates among hematologic malignancies could have influenced the observed associations.

We acknowledge your suggestion regarding the potential value of analyzing the association between BMI and the incidence of hematological malignancies. We are aware that the JACC Study includes incidence data; however, these data are limited to certain geographic areas and thus pertain to a subset of participants different from those included in the present analysis. Due to these differences in the study population and data availability, incorporating incidence analyses within this manuscript was not feasible.

Nonetheless, we recognize the importance of this research question and plan to address it in future studies using the incidence data. We have added the statement in the Conclusion section to highlight future research directions.

In summary, we have revised the Discussion and the Conclusion section to more explicitly address the limitations and implications of using mortality as the primary outcome, particularly in relation to hematological malignancies with favorable prognosis such as certain lymphoma subtypes. We appreciate your valuable input, which has helped us to strengthen the contextual interpretation of our findings and to outline clear avenues for future investigation.

Change

(page 4)

*Especially, cancer mortality is one of the most robust and key indices, as it reflects not only the incidence of cancer but also biological and clinical outcomes, as well as social factors such as access to medical and health care. Because mortality can be influenced by such social factors, it is important to focus on this outcome. Furthermore, according to the Ministry of Health, Labour, and Welfare, cancer mortality has been set as a key indicator ever since the establishment of the Basic Plan to Promote Cancer Control Programs [22–25]. Moreover, in the Basic Plan to Promote Cancer Control Programs (Phase 4), poor-prognosis cancer mortality is included as one of the indicators, although no explicit definition is provided in the plan. In practice, however, a 5-year survival rate of less than 50% [25–27] is sometimes used as a criterion for defining poor-prognosis cancers, which include leukemia and multiple myeloma [28–30]. Given these considerations, focusing on cancer mortality remains essential for understanding and addressing the overall cancer burden.*

(page 18)

*While the present study focused on mortality as the outcome, future studies should investigate the association between BMI and cancer incidence in the Japanese population to provide a more comprehensive understanding of their relationship.*

(page 16)

*Furthermore, analysis of data from 2000–2008 revealed notable differences in the 5-year survival rates, with lymphoma showing 54.6–65.5%, compared with 29.0–36.4% for multiple myeloma and 32.1–39.2% for leukemia [28-30].*

2. BMI was measured only at baseline. Please clarify whether any sensitivity analyses were conducted to account for potential weight changes over time or reverse causation, particularly given the long duration of follow-up.

Author response

Thank you for your important comment.

Body weight and height were measured only at baseline, and no follow-up data on these variables were available, making sensitivity analyses accounting for weight changes over time unfeasible.

However, a study conducted among Japanese showed that the weight tends to be relatively stable in the middle to older adult population. Thus, the effect of changing weight might not be crucial on our results.

In addition, to address potential reverse causation, we have already excluded participants with less than five years of follow-up from the analysis. This approach reduces the likelihood of bias caused by weight loss or gain due to preclinical disease.

We have added a statement to the Discussion section to acknowledge the absence of longitudinal weight data as a limitation of the study.

Change

(page 17)

*Additionally, body weight and height were measured only at baseline, and no information on weight changes during follow-up was available. Therefore, we could not assess the potential impact of weight change over time on the association between BMI and mortality. Nevertheless, this may not critically affect our results, as previous Japanese epidemiological study have shown that the body weight of middle-aged and older adults tends to be relatively stable [53]. Moreover, participants with less than five years of follow-up were excluded to minimize reverse causation due to preclinical disease, residual bias from unmeasured weight changes cannot be completely ruled out.*

3. The cause of death was determined from death certificates, which may lack diagnostic specificity, especially for subtypes of hematological malignancies. Please consider adding a discussion on the potential for misclassification of cancer subtypes.

Author response

Thank you for your insightful feedback. We appreciate your point regarding the potential for misclassification of hematological malignancies due to the use of death certificates.

We have added a discussion to address this potential limitation. We acknowledge that the lack of diagnostic specificity in death certificates could theoretically lead to misclassification of cancer subtypes. However, we also cite a previous study that reported a high concordance rate (81%) between autopsy results and death certificates for cancer deaths. Based on this evidence, we believe that any misclassification would not significantly impact our study's main conclusions. This new addition clarifies the potential limitation while providing evidence to support the validity of our findings.

Change

*(page 16)*

*Fourth, due to using data from the death certificates, misclassification might occur for the death causes. However, a previous study found that the concordance between autopsy results and the cause of death on the death certificates was relatively high for cancer (81%). Therefore, it will not significantly affect our results [52].*

4. Smoking is an important risk factor for hematological malignancies and is also closely associated with BMI. Therefore, conducting analyses stratified by smoking status or evaluating potential interactions between smoking and BMI would be informative.

Author response

Thank you for the valuable suggestion regarding stratification by smoking status and evaluation of potential interactions between smoking and BMI.

We conducted stratified analyses by smoking status to explore this; the results showed a similar trend of increased hazard ratios for obesity. However, due to the relatively small number of deaths in some strata, the confidence intervals became wide, and in certain models, convergence issues arose. These factors made interpretation difficult. The attached figure is provided for your reference, showing the results up to Model 2. As you can see, the trend remains consistent even with the stratification.

Therefore, we decided to present the overall results while acknowledging this limitation in the Discussion section.

Change

(page 17)

*Finally, the stratified analysis, which would have allowed us to evaluate the influence of important covariates such as sex and smoking, was not feasible due to the insufficient number of mortalities.*

5. Were there any differences by sex? Given possible sex differences in BMI distribution and hematologic cancer risk, stratified analyses by sex would also be valuable.

Author response

Thank you for the valuable suggestion regarding stratification by sex.

As with the smoking-stratified analyses, we made a concerted effort to conduct stratified analyses by sex to explore this. The results consistently showed a similar trend of increased hazard ratios for obesity in both men and women. However, as in the smoking-stratified analyses, the relatively small number of deaths in some strata led to wide confidence intervals, and in certain models, convergence issues arose. These factors made reliable interpretation challenging. The attached figure is provided for your reference, showing the results up to Model 2. As you can see, the trend remains consistent even with the stratification.

Therefore, we decided to present the overall results while clearly acknowledging this limitation in the Discussion section.

Change

(page 17)

*Finally, the stratified analysis, which would have allowed us to evaluate the influence of important covariates such as sex and smoking, was not feasible due to the insufficient number of mortalities.*

Reviewer 2

1 Is this study assessing association between obesity in 1990 and mortality in later period among patients suffering from hematological malignancies? Mortality can be affected by many other factors such as renal dysfunction, diabetes, hyperlipidemia, hypertension, arteriosclerosis, etc. Without assessing these factors, the true association between obesity in about 20 years ago and mortality in later life is impossible to clarify. As the authors wrote in line 203, renal dysfunction, diabetes, and arteriosclerosis, etc, negatively affect treatment outcomes. Therefore, just assessing obesity in the past and mortality is meaningless.

Instead, the author should explore an association between obesity and incidence of hematological malignancy in later period. CHIP theory may demonstrate that obese people may have an increased incidence of hematological malignancy.

Author response

Thank you for your careful review and insightful comments. We appreciate you raising these important points, particularly the role of potential confounders and the study's scope.

Regarding your first point, we agree that various clinical factors such as renal dysfunction, diabetes, hyperlipidemia, hypertension, and arteriosclerosis can significantly influence mortality in patients with hematological malignancies. However, in our conceptual framework, these conditions can be considered as the key mediators in the causal pathway between obesity and mortality. Obesity is a well-established risk factor for these comorbidities, and these conditions, in turn, can compromise treatment tolerance and long-term survival. Adjusting for these factors would, therefore, lead to an underestimation of the total effect of obesity on mortality [R2].

Regarding the concern about using past BMI data, we have referenced previous Japanese epidemiological studies that show body weight and BMI tend to be relatively stable in the adult population. This suggests that BMI from the early 1990s is a reasonable proxy for long-term obesity exposure in our study cohort. We have added about this limitation in the discussion section.

We also respectfully disagree with the suggestion to focus solely on the incidence of hematological malignancies. Mortality is a critical endpoint because obesity’s impact extends beyond cancer incidence to treatment response, toxicity, recurrence, and overall survival. Mortality reflects the integrated biological and clinical consequences of obesity—including effects on immune function, pharmacokinetics, and comorbidities—which are highly relevant after diagnosis. Moreover, in Japan’s national cancer control policy, reduction of cancer mortality is explicitly identified as a major public health goal [R1]. Providing robust evidence on modifiable risk factors for cancer mortality is therefore essential for developing effective prevention and intervention strategies that can guide policy and practice. We have added a statement in the Conclusion section to highlight future research directions

We acknowledge that investigating the incidence of hematological malignancies is also important. However, such analyses require a more restricted subset of participants and are beyond the scope of the present study. We plan to address this topic in future research. We have added the statement in the Conclusion section to highlight future research directions.

We hope these clarifications address your concerns and underscore the importance of our focus on mortality as the primary outcome.

Change

(page 4)

*Especially, cancer mortality is one of the most robust and key indices, as it reflects not only the incidence of cancer but also biological and clinical outcomes, as well as social factors such as access to medical and health care. Because mortality can be influenced by such social factors, it is important to focus on this outcome. Furthermore, according to the Ministry of Health, Labour, and Welfare, cancer mortality has been set as a key indicator ever since the establishment of the Basic Plan to Promote Cancer Control Programs [22–25]. Moreover, in the Basic Plan to Promote Cancer Control Programs (Phase 4), poor-prognosis cancer mortality is included as one of the indicators, although no explicit definition is provided in the plan. In practice, however, a 5-year survival rate of less than 50% [25–27] is sometimes used as a criterion for defining poor-prognosis cancers, which include leukemia and multiple myeloma [28–30]. Given these considerations, focusing on cancer mortality remains essential for understanding and addressing the overall cancer burden.*

(page 18)

*While the present study focused on mortality as the outcome, future studies should investigate the association between BMI and cancer incidence in the Japanese population to provide a more comprehensive understanding of their relationship.*

2 In Figure 1, overweight group shows a lowered risk though they are not statistically significant. CHIP and BMAT theory cannot explain the opposite trend. How do you explain?

Author response

Thank you for your valuable comment. You are correct that our results showed no significant association between the overweight group and mortality from hematological malignancies.

First, a previous study reported that Clonal Hematopoiesis of Indeterminate Potential (CHIP), a potential mediator between obesity and hematological malignancies, tended to be less prevalent in overweight individuals compared to those who were obese. This is not contradictory to our results that only participants with obesity had a higher risk for hematological malignancy death. To clarify this, we revised the sentence regarding the association between obesity and CHIP in the Discussion section.

In addition, basically BMAT is associated with BMI; however one previous study could not detect the difference in BMAT between normal and obese people [R3] . This might mean that BMAT and BMI are associated but not particularly strong. This could mean that the biological effects mediated by BMAT become pronounced only at the higher BMI levels characteristic of obesity. Moreover, in our participants, the mean BMI was 22.03 ± 1.68 in normal weight group, 26.63 ± 1.25 in overweight group and 32.99 ± 13.66 in obese group. To provide more context, we have also added the mean BMI values for each group to Table 1 in the revised manuscript.

In summary, we do not interpret the lack of a significant association in the overweight group as a lower risk. Rather, it suggests that the increase in mortality risk becomes substantial only at the higher BMI levels found in the obese group.

Change

(page 14)

*Thus, the risk of the presence of CHIP is the highest for those with obesity.*

(Table 1)

Added the mean BMI and standard deviations.

3 In Figure 1, if the authors included covariates such as age, sex, drinking status etc in model 3, all HRs and 95% CIs should be shown. Is there any other variable associated with the mortality of hematological malignancies?

Author response

We thank the reviewer for this valuable suggestion.

The primary aim of our study was to examine the association between BMI and mortality from hematological malignancies. In Model 3, covariates such as age, sex, drinking status, and other factors listed in the Methods section were included solely to control for potential confounding. Their individual hazard ratios were not the focus of our research question.

Presenting HRs and 95% CIs for all covariates could dilute the emphasis on BMI, which is the main exposure of interest in this study. In addition, previous studies have noted that presenting estimates for confounders in such contexts can sometimes be misleading and make interpretation more difficult [R4,R5]. Consistent with common practice in epidemiological research, we therefore reported only the HRs for the main exposure variables.

To ensure clarity and transparency, we have now explicitly listed all covariates adjusted for in Model 2 and Model 3 within the figure itself, in addition to the full descriptions provided in the Methods and figure legends.

Change

We have now clearly indicated the covariates adjusted for each model at the bottom of Figure 2.

4 The average age (SD) or age group must be shown. Age has the strongest impact on mortality in general. By the way, I cannot find Table 1.

Author response

We appreciate your comment. The age group distribution was already presented in Table 1 (originally as the second item, now as the fourth item after revision). To enhance clarity and assist readers in locating this information, we have added a sentence in the Results section referring explicitly to Table 1, and we have also added the mean age and standard deviations to Table 1. To ensure the table is easily accessible, we have relocated Table 1 to its appropriate position within the Results section.

Change

(page 9)

*The baseline characteristics, including sex, age, and other demographic variables, of the 97,073 participants (40,479 men (41.7%) and 56,594 women (58.3%)) are presented in Table 1. A total of 1,671 participants (1.7%) were classified as obese.*

(Table1)

Added the mean age and standard deviations.

References

R1.	Japan. Cancer Control Act (GAN TAISAKU KIHON HOU). 2006 [cited 10 Aug 2025]. Available: https://laws.e-gov.go.jp/law/418AC1000000098

R2.	Schisterman EF, Cole SR, Platt RW. Overadjustment Bias and Unnecessary Adjustment in Epidemiologic Studies. Epidemiology. 2009;20: 488–495. doi:10.1097/EDE.0b013e3181a819a1

R3.	Veldhuis-Vlug AG, Rosen CJ. Clinical Implications of Bone Marrow Adiposity. J Intern Med. 2018;283: 121–139. doi:10.1111/joim.12718

R4.	Hünermund P, Louw B. On the Nuisance of Control Variables in Causal Regression Analysis. Organizational Research Methods. 2023;28: 138–151. doi:10.1177/10944281231219274

R5.	Westreich D, Greenland S. The Table 2 Fallacy: Presenting and Interpreting Confounder and Modifier Coefficients. Am J Epidemiol. 2013;177: 292–298. doi:10.1093/aje/kws412
