# Supplementary material for: Association between obesity and mortality from hematological malignancies among Japanese adults: The Japan Collaborative Cohort study
Source: PLoS One. 2025 Oct 30;20(10):e0335750. doi: 10.1371/journal.pone.0335750 (PMC12574875; doi:10.1371/journal.pone.0335750)
Supplement: Attachment — Submitted filename: response to reviewers_20250926.docx [file pone.0335750.s004.docx]

Revision Note

We are most grateful to you and the reviewers for the comments on our manuscript. These have greatly helped us to improve our manuscript.　We have revised our manuscript based on the constructive suggestions of the reviewers.

The following are our responses to the reviewer 1 and 2. Our responses are in blue, and the changes are in italics. Also, the changes are shown in red in the revised manuscript.

Reviewer 1

1. The authors have appropriately addressed all of my comments, and I sincerely thank them for their careful and thoughtful revisions.

Author response

We would like to express our sincere gratitude for your thoughtful and constructive feedback throughout the review process. Your kind words and valuable suggestions have been instrumental in improving the quality of our work.

Reviewer 2

1. I understand that clinical diseases such as DM and renal dysfunction may be the key mediators in the causal pathway. Although the authors are applying the full model in model 3 in Figure 3 (traditional methods), not causal regression analysis for each dependent variable, I think that is OK. I think full adjusted model (traditional) is better than the causal regression analysis to interpret the results. Because causal analysis framework can vary largely depending on researchers, it causes large difference in OR and RR etc. So, I understand your explanation, not adjusting clinical diseases.

Author response

We sincerely appreciate your generous understanding and for your thoughtful evaluation of our approach. Your supportive comments are greatly appreciated.

2. In figure 2, the authors must show all HRs with age, sex, and other variables. Although the authors explained that “presenting all HRs for all covariates could dilute the emphasis on BMI,” the journal readers do not focus on the only BMI. We want to see the strength of all risk factors the authors included. Therefore, we can interpret the results of BMI on mortality.

Besides, concealing the other HRs in manuscript can lead to salami publication. To avoid it, the authors must present all HRs in any cases. If there is a problem in volume, how about creating supplementary tables.

Author response

We sincerely thank the reviewer for this important and constructive comment. In response, we have now included all HRs for age, sex, and other covariates in the Supplementary Table, as suggested.

During this process, we also carefully reconsidered the categorization of several covariates　(childhood living area, educational attainment, and employment status) and made minor adjustments to improve clarity and consistency. Importantly, these modifications did not have any meaningful impact on the results or on the interpretation of BMI’s association with mortality.

Change

(Page 8)

*childhood living area (large cities, other cities, rural areas/islands or remote areas, and others), educational attainment (<10 years, <13 years, <16 years, and ≥16 years), employment status at baseline (employed/part-time, self-employed, homemaker, unemployed, and others),*

(Page 10)

Table 1 has been revised accordingly to correspond to the changes in covariate categorization.

(Page 2)

*Compared with normal-weight individuals, those classified as obese exhibited a significantly higher risk of mortality from all hematological malignancies (HR: 1.78; 95% CI: 1.02–3.11), multiple myeloma (HR: 2.75; 95% CI: 1.09–6.94), leukemia (HR: 2.47; 95% CI: 1.07–5.69), and particularly myeloid leukemia (HR: 3.89; 95% CI: 1.66–9.11).*

(Page 12)

*In disease-specific subgroup analyses, obesity was significantly associated with higher mortality from multiple myeloma (HR: 2.75, 95% CI: 1.09–6.94), leukemia (HR: 2.47, 95% CI: 1.07–5.69), and particularly myeloid leukemia (HR: 3.89, 95% CI: 1.66–9.11) in Model 3.*

(Fig. 2)

Fig. 2 has been revised accordingly to correspond to the changes in covariate categorization.

(S1 Table)

HRs for all variables included for each model are shown in the supporting information, S1 Table. For convenience, we have included the S1 Table below.

3. in Figure 2, as for time at risk (person-years), the numbers in 4 body weight group are all the same in all analyses (malignancies, lymphoma, MM, leukemia, and Myeloid leukemia. i.e: 84382, 1211682, 327241, 28133). Generally, mortality rate should be exclusive by diseases. For instance, what happens to an obese person who died from multiple myeloma? How was the patient handled in leukemia analysis? Was the patient obese and he/she survived in leukemia analysis?
In leukemia analysis, patients who died from lymphoma, MM, myeloid leukemia should be excluded from the beginning.

Author response

We sincerely thank the reviewer for raising this important point. Generally, in disease-specific survival analyses, deaths from other causes are treated as censored observations. Accordingly, in our analyses, we applied the same approach. For example, in the multiple myeloma analysis, deaths from other hematologic malignancies such as lymphoma and leukemia, as well as deaths from non-hematologic causes, were censored, and thus, person-years were the same in all analyses.

To clarify this procedure, we have added a description to the Methods section to explicitly state how other causes of death were handled in each disease-specific analysis.

Change

(Page 9)

*For cause-specific analyses, deaths from causes other than the outcome of interest were treated as censored at the time of death.*
